# Burden trend and forecasting analysis of malignant skin melanoma from 1990 to 2021

**DOI:** 10.1097/JS9.0000000000002908

**Published:** 2025-07-24

**Authors:** Xingkai Wang, Junwu Huang, Jianping Hu, Xiaolong Ma, Zhen Huang, Jiazhuang Zhu, Chunlin Zhang, Kunpeng Zhu

**Affiliations:** a Department of Orthopaedics, Shanghai Tenth People’s Hospital, Tongji University, School of Medicine, Shanghai, China; b Institute of Bone Tumor, Tongji University, School of Medicine, Shanghai, China; c Department of Orthopaedics, Jinshan Branch of Shanghai Sixth People’s Hospital, Shanghai, China

**Keywords:** disability-adjusted life-years, global burden of disease, incidence, malignant skin melanoma, prevalence

## Abstract

Malignant skin melanoma (MSM) is a highly malignant skin tumor that poses a significant threat to public health worldwide owing to its high mortality and susceptibility to metastasis. We aimed to conduct a comprehensive, multifaceted, and methodical analysis of the burden trends regarding MSM patients across all ages. We acquired the prevalence, incidence, and disability-adjusted life year data of all ages and age-standardized patients from 1990 to 2021 worldwide, spanning 204 nations, 21 regions, and 5 SDI areas, using the Global Burden of Disease database. Additionally, we employed joinpoint regression analysis to get the average annual percentage changes (for the aforementioned metrics. Lastly, by combining data from several levels, we examined trends in MSM burden. In addition, we first predict the general trend of MSM over the next 15 years using the autoregressive integrated moving average model. Based on the above studies, since the 1990s, the worldwide health burden of MSM has been steadily increasing. Across levels and populations, the burden of MSM varies significantly. More effective and targeted interventions need to be developed as a means of managing the MSM burden, thereby alleviating global public health pressures.

## Introduction

Malignant melanoma is a highly malignant tumor of melanocyte origin that occurs primarily in the skin^[1]^. Approximately 6.8–20% of cutaneous malignant tumors are malignant skin melanoma (MSM), making it the third most prevalent type^[2]^. It occurs mainly in adults, predominantly Caucasians, with a lower incidence in dark-skinned people, and extremely rarely in children^[2–4]^. Chronic UV exposure and family history are the major underlying pathogenic factors for MSM^[5–7]^. Research has indicated that MSM is intimately linked to immune escape mechanisms and gene mutations like BRAF and NRAS^[5]^. The early stages of MSM are characterized by irregular margins, pigmentation, and asymmetrical skin lesions.

**HIGHLIGHTS**

- Age-standardized incidence and prevalence of malignant skin melanoma (MSM) show an increasing trend between 1990 and 2021, while the age-standardized rate of disability-adjusted life years decreases.
- The global burden of MSM is higher for males than females in the period 1990–2021.
- MSM burdens are higher in high SDI areas than in areas with low indices, and SDI is positively correlated with MSM.
- The global MSM burden is forecast to generally decline over the next 15 years.

Surgery may result in a better prognosis for people with early MSM. The absence of specialized therapy and the dismal prognosis of individuals with intermediate and severe illness, however, make early identification and treatment of MSM crucial^[8]^. Furthermore, MSM is responsible for over 90% of skin cancer fatalities, and given its high lethality, it poses a significant challenge to healthcare professionals worldwide^[9]^. The prevalence of MSM has been rising worldwide over the last three decades, partly as a result of changing lifestyles and increasing UV exposure brought about by climate change^[10,11]^. This has had detrimental effects on society. Consequently, a thorough grasp of the health burden of MSM is necessary and contributes significantly to the creation of focused therapies.

Details of MSM incidence, prevalence, and disability-adjusted life years (DALYs) in various nations, regions, and socio-demographic index (SDI) levels between 1990 and 2021 are available in the recently updated Global Burden of Disease (GBD) 2021 database. In order to investigate and evaluate the health burden of MSM across various nations and regions, we retrieved all of their data for this study. At the same time, we categorized them based on gender and SDI levels and evaluated the disease burden and trends at various levels.

This study complies with the reporting requirements of the Transparency In The reporting of Artificial INtelligence – the TITAN guideline^[12]^. As it does not involve AI technology, relevant statements are detailed in the methodology section.

## Methods

### Artificial intelligence usage statement

This study did not utilize any artificial intelligence (AI) technology to assist with research design, data processing, result analysis, or manuscript writing. All statistical analyses were performed using R software (version 4.3.2), and data interpretation and conclusion drawing were conducted independently by the research team without the involvement of generative AI or other AI tools.

### Data collection

The target population for this study was derived from the Global Burden of Disease Study 2021 dataset, which included all MSM cases, according to the Global Health Data Exchange (Homepage|Institute for Health Metrics and Evaluation) platform. In May 2024, the Global Burden of Disease Study was completely revised to incorporate the worldwide burden of 87 risk factors and 369 illnesses and injuries for 204 nations and 21 regions from 1990 to 2021^[13,14]^. Prevalence, incidence, and DALYs regarding MSM, as well as age-standardized data of these indicators, were gathered for the current analysis from 204 nations, 21 regions, and several SDI areas, including all age groups. It is possible to reduce the impact of variations in the age distribution of inhabitants among nations on the outcomes by using age standardization^[15]^. The World Health Organization’s (WHO) definition of standard populations served as the basis for age standardization in GBD research, which makes data from various nations or areas more internationally comparable. DALYs, which are designed to capture the overall burden of an illness, injury, or health condition on an individual and society through a single numerical value, are defined as the total healthy life years lost from morbidity to mortality, including life years lost due to premature death and healthy life years lost due to disability^[16]^. One of the frequently employed indicators in GBD research is DALYs, a composite indicator that quantifies the effect of illness on human health. A composite measure of a nation’s level of development, the SDI, is determined by taking into account factors including fertility rate, average educational attainment, and per capita income^[17]^. At a minimum of zero, the country’s level of development is better when the SDI is closer to 1^[13]^. The GBD database categorizes 204 nations into five levels based on SDI, namely high SDI (41 nations), high-middle SDI (41 nations), middle SDI (40 nations), low-middle SDI (41 nations), and low SDI (41 nations)^[18]^.

### Statistical analysis

National and international health surveys, clinical studies and hospital records, death records and registries, literature, and systematic reviews constitute various data sources utilized in the GBD database. Prevalence and incidence estimates derived from Bayesian modeling were provided by the GBD database, aiding in the precise evaluation of the health burden. In addition, the GBD database provided 95% uncertainty intervals (UIs) for the estimates, defined by the 25th and 975th values out of 1000 ordered estimates. To better appreciate the uncertainty in the data estimates, this includes age-standardized rates as well as rates per 100 000 persons for all ages. Using joinpoint software, we performed joinpoint regression analysis to determine the annual percent change (APC), average annual percent change (AAPC), and 95% confidence interval (CI) for the globe, various genders, five SDI regions, and 21 districts^[19]^. Over subperiods of time, linear trends in prevalence, incidence, and DALYs were evaluated using APC^[20]^. Utilizing the weighted average of the APC, the AAPC evaluates the average change throughout the whole period^[21]^. For data analysis and visualization, we used R (version 4.3.2) and the joinpoint regression application (version 5.0.2). The autoregressive integrated moving average (ARIMA) model consists of an autoregressive (AR) model and a moving average (MA) model based on the “forecast” R package. The threshold for statistical significance was set at 0.05. The AAPC calculation formula is shown below (*a_i_* is the slope coefficient for the *i*th segment with *i* indexing the segments in the desired range of years, and *w_i_* is the length of each segment in the range of years):

$$AAPC=\left\{exp\left(\frac{\sum w_{i}b_{i}}{\sum w_{i}}\right)-1\right\}\times 100$$

## Results

### Global trends in MSM

**Figure 1.**
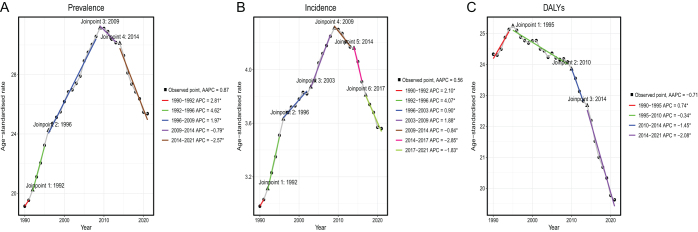
Joinpoint regression analysis of malignant skin melanoma from 1990 to 2021. (A) Age-standardized prevalence rate of MSM. (B) Age-standardized incidence rate of MSM. (C) Age-standardized DALY rate of MSM. DALY, disability-adjusted life year; MSM, malignant skin melanoma.

Analyzing globally, prevalence increased from 833 215.7 (95% UI 813 313–849 960.7) in 1990 to 217 7566.1 (95% UI 205 7878.7–2 274 067.9) in 2021 for MSM (Table 1). With an AAPC of 0.87 (95% CI 0.68–1.06), the age-standardized prevalence rates (ASPRs) for MSM per 100 000 population were 19.1 (95% UI 18.7–19.5) in 1990 and 25.4 (95% UI 24.0–26.5) in 2021. The prevalence of MSM peaked in 2010 and then declined, although overall, it continued to rise (Fig. 1A). In terms of incidence numbers analyzed, in 2021, the number was 303 104.6 (95% UI 281 717.6–318 904.8), up from 124 319.8 (95% UI 119 603.9–127 610.5) in 1990 (Supplemental Digital Content, Table S2, available at: http://links.lww.com/JS9/E781). Meanwhile, the age-standardized incidence rate (ASIR) increased from 3.0 (95% UI 2.9–3.1) per 100 000 population in 1990 to 3.6 (95% UI 3.3–3.7), with an AAPC of 0.56 (95% CI 0.33–0.79). Consistent with the trend in prevalence, there was a peak in MSM in 2010, with a subsequent decline but an upward trend overall (Fig. 1B). Additionally, the number of DALY cases in MSM worldwide increased from 1990 to 2021, reaching 1 045 777.5 (95% UI 959 373.2, 1 103 849) and 1 678 836.3 (95% UI 1 474 533.7, 1 837 368.8), respectively (Supplemental Digital Content Table S3, available at: http://links.lww.com/JS9/E782). Yet among MSM, the age-standardized rate of DALYs dropped from 24.3 (95% UI 22.4–25.6) to 19.6 (95% UI 17.2–21.5). Global DALYs for MSM exhibit a distinct decreasing trend between 1990 and 2021, with an AAPC of −0.71 (95% CI −0.82 to −0.60) (Fig. 1C).

**Table 1:** Cases, age-standardized prevalence rates, and AAPC of malignant skin melanoma in 1990–2021

	Prevalence (95% UI)				
	Cases in 1990	Age-standardized rate in 1990 (per 100 000)	Cases in 2021	Age-standardized rate in 2021 (per 100 000)	AAPC (95% CI)
Global	833 215.7 (813 313.0–849 960.7)	19.1 (18.6–19.5)	2 177 566.1 (2 057 878.7–2 274 067.9)	25.4 (24.0–26.5)	0.87 (0.68–1.06)
Gender					
Male	392 702.8 (383 550.0–400 565.3)	19.1 (18.7–19.5)	1 124 642.1 (1 067 818.0–1 183 251.2)	27.8 (26.3–29.3)	1.19 (0.93–1.45)
Female	440 512.9 (426 448.9–452 666.7)	19.4 (18.7–19.9)	1 052 924.0 (982 868.7–1 122 204.9)	23.6 (22.1–25.2)	0.62 (0.39–0.85)
SDI level					
High	694 826.2 (679 053.4–708 762.2)	67.2 (65.7–68.5)	1 587 032.5 (1 504 224.6–1 640 716.4)	91.4 (87.7–94.2)	0.98 (0.70–1.26)
High-middle	117 156.0 (112 354.9–121 553.9)	11.0 (10.6–11.5)	448 236.7 (402 261.7–482 343.3)	24.6 (22.0–26.4)	2.65 (2.41–2.88)
Middle	14 235.5 (10 799.5–15 996.5)	1.0 (0.8–1.1)	107 089.9 (76 731.5–127 488.7)	3.8 (2.8–4.6)	4.38 (4.20–4.57)
Low-middle	3956.2 (2840.3–4918.9)	0.5 (0.3–0.6)	22 555.3 (15 546.7–27 934.7)	1.3 (0.9–1.6)	3.27 (3.17–3.38)
Low	2417.8 (1503.5–3133.3)	0.7 (0.5–1.0)	10 425.4 (6106.5–14 369.6)	1.3 (0.8–1.7)	1.81 (1.68–1.94)
GBD region					
Andean Latin America	353.2 (275.9–454.4)	1.4 (1.1–1.8)	3515.2 (2608.1–4778.4)	5.6 (4.1–7.5)	4.71 (4.10–5.33)
Australasia	50 522.5 (47 798.7–53 523.9)	222.8 (210.9–236.4)	122 143.9 (110 343.2–134 052.7)	266.8 (244.7–289.6)	0.62 (−0.13–1.37)
Caribbean	704.4 (658.3–753.5)	2.4 (2.2–2.6)	3018.2 (2637.8–3444.3)	5.7 (5.0–6.5)	3.02 (2.23–3.80)
Central Asia	1889.3 (1717.1–2082.1)	3.5 (3.2–3.9)	4349.4 (3757.8–4999.3)	4.7 (4.1–5.4)	0.91 (0.03–1.80)
Central Europe	23 751.3 (22 515.0–25 923.3)	16.6 (15.7–18.1)	92 711.3 (82 247.5–102 940.1)	51.9 (46.0–57.3)	3.77 (3.57–3.98)
Central Latin America	1736.9 (1683.9–1805.0)	1.5 (1.5–1.6)	16 323.2 (14 485.4–18 233.5)	6.2 (5.5–6.9)	4.70 (4.00–5.40)
Central sub-Saharan Africa	186.6 (129.1–293.0)	0.6 (0.4–0.9)	902.7 (604.6–1567.9)	1.1 (0.7–1.8)	1.86 (1.71–2.02)
East Asia	7486.9 (4771.5–9350.0)	0.7 (0.4–0.8)	82 178.1 (43 901.5–110 985.2)	4.1 (2.2–5.5)	6.05 (5.74–6.37)
Eastern Europe	35 987.3 (34 336.4–38 577.0)	13.7 (13.1–14.7)	126 651.5 (116 335.8–136 904.9)	42.2 (38.7–45.6)	3.87 (3.06–4.69)
Eastern sub-Saharan Africa	1636.6 (1047.4–2067.9)	1.4 (0.9–1.8)	7289.8 (4048.1–11 269.1)	2.4 (1.4–3.6)	1.80 (1.66–1.93)
High-income Asia Pacific	12 620.5 (11 828.3–13 441.4)	6.3 (5.9–6.7)	47 370.3 (39 682.4–52 958.9)	15.0 (12.5–16.7)	2.84 (2.24–3.46)
High-income North America	430 579.8 (418 238.5–439 060.6)	132.8 (129.4–135.5)	758 702.6 (719 677.4–784 585.5)	133.3 (127.3–137.5)	−0.00 (−0.43–0.42)
North Africa and Middle East	5536.3 (2782.9–7901.6)	2.3 (1.2–3.3)	61 197.2 (33 273.2–75 784.9)	11.4 (6.1–14.2)	5.26 (5.07–5.44)
Oceania	10.0 (7.0–16.9)	0.3 (0.2–0.5)	25.9 (18.1–40.2)	0.3 (0.2–0.4)	0.04 (−0.05–0.12)
South Asia	2866.4 (1915.0–3753.5)	0.3 (0.2–0.5)	18 079.2 (11 304.0–26 012.4)	1.0 (0.6–1.4)	3.55 (3.43–3.66)
Southeast Asia	775.8 (588.4–1104.3)	0.2 (0.2–0.3)	3038.4 (2115.7–4211.6)	0.4 (0.3–0.6)	2.08 (1.98–2.18)
Southern Latin America	2475.3 (2333.8–2624.8)	5.2 (4.9–5.5)	14 240.5 (13 293.2–15 325.0)	17.8 (16.7–19.2)	4.14 (3.69–4.59)
Southern sub-Saharan Africa	954.7 (706.2–1332.1)	2.6 (1.9–3.6)	3505.4 (2067.7–5067.4)	4.8 (2.8–6.7)	2.05 (1.57–2.53)
Tropical Latin America	3867.2 (3720.8–4037.2)	3.1 (3.0–3.3)	21 717.8 (20 715.9–22 710.3)	8.3 (7.9–8.7)	3.13 (2.58–3.69)
Western Europe	248 538.1 (241 100.0–255 821.3)	51.7 (50.2–53.0)	787 257.5 (742 327.5–821 229.0)	113.0 (108.2–117.2)	2.60 (2.23–2.98)
Western sub-Saharan Africa	736.2 (335.7–971.9)	0.6 (0.3–0.8)	3348.0 (1109.9–5016.9)	1.0 (0.4–1.5)	1.71 (1.56–1.86)

Taken together, the global prevalence and incidence of MSM increased significantly from 1990 to 2021, with a generally upward trend, despite a decline after peaking in 2010. At the same time, despite an increase in the total number of DALY cases, the rate of age-standardized DALYs has continued to decline, which may be attributed to demographic changes and improved medical care.

### Global trends by gender in MSM

**Figure 2.**
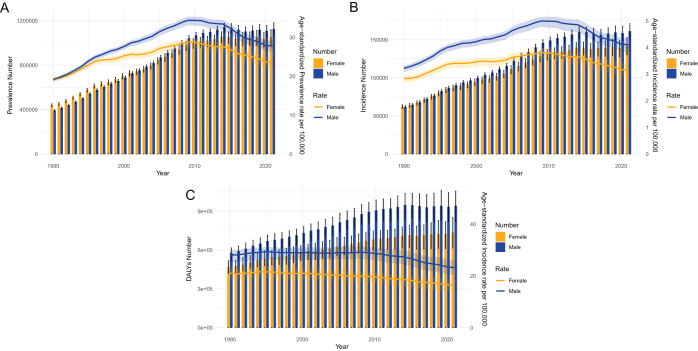
The number and age-standardized rates of prevalence (A), incidence (B), and DALYs (C) of malignant skin melanoma globally between males and females from 1990 to 2021. DALY, disability-adjusted life year.

Age-standardized prevalence and incidence rates of MSM in males and females grew considerably worldwide between 1990 and 2021, but DALY rates had a sharp decline with notable gender-specific heterogeneity (Fig. 2A–C). Compared to females, who had an ASPR of 23.6 (95% UI 22.1–25.2) per 100 000 population in 2021, males had a higher rate of 27.8 (95% UI 26.3–29.3) per 100 000 (Table 1). Furthermore, males observed a considerably greater increasing trend in ASPRs than females (ASPR = 1.19, 95% UI 0.93–1.45 vs. ASPR = 0.62, 95% UI 0.39–0.85). In line with the trend in ASPRs, males (ASIR = 4.1/100 000, 95% UI 3.8–4.4) demonstrated a far greater increasing trend than females (ASIR = 3.2/100 000, 95% UI 2.9–3.4), with AAPCs of 0.77 (95% CI 0.50–1.04) and 0.33 (95% CI 0.50–1.04), respectively (Supplemental Digital Content, Table S2, available at: http://links.lww.com/JS9/E781). Nonetheless, both males and females exhibited a declining trend in age-standardized DALY rates, with the reduction being larger for females than for males (Supplemental Digital Content Table S3, available at: http://links.lww.com/JS9/E782). Even so, males had greater age-standardized DALY rates than women, with 23.2 (95% UI 20.3–26.0) and 16.6 (95% UI 13.8–19.2) per 100 000 population, respectively. In conclusion, males had a larger burden of MSM than females, as evidenced by the fact that the rates of age-standardized incidence, prevalence, and DALYs of MSM were higher in males than in females between 1990 and 2020, both in terms of alterations and absolute values.

### Regional trends in MSM

In 2021, Western Europe was the region with the highest number of patients at 787 257.5 (95% UI 742 327.5–821 229.0), followed by high-income North America at 758 702.6 (95% UI 719 677.4–784 585.5), and the lowest was in Oceania at 25.9 (95% UI 18.1–40.2). In a similar vein, the incidence and DALYs were highest in Western Europe (100 441.7; 95% UI 94 012.4–105 087.2) and lowest in Oceania (23.9). Age-standardized rates of incidence (32.4/100 000, 95% UI 29.6–35.2), DALYs (110.0/100 000, 95% UI 100.6–120.6), and prevalence (266.8/100 000, 95% UI 244.7–289.6) were highest in Australasia. Oceania, on the other hand, has the lowest ASPRs, at 0.3 per 100 000 (95% UI 0.2–0.4). Both the ASIRs (0.2 per 100 000, 95% UI 0.2–0.3) and the DALY rates (5.4 per 100 000, 95% UI 3.7–7.1) were lowest in Southeast Asia. Prevalence and incidence (except in Oceania) are on the rise in all regions except high-income North America. In terms of incidence and prevalence, the fastest-growing regions were Eastern Europe (AAPC: 3.03, 95% CI 2.34–3.72) and East Asia (AAPC: 6.05, 95% CI 5.74–6.37). The fastest-growing and fastest-declining DALY rates were seen in Eastern Europe and Australasia, respectively, with lesser changes in DALY rates observed in the remaining areas. All data were presented in Table 1, Supplemental Digital Content Table S1, available at: http://links.lww.com/JS9/E780, Supplemental Digital Content Table S2, available at: http://links.lww.com/JS9/E781, and Supplemental Digital Content Figure S1, available at: http://links.lww.com/JS9/E627. Above all, the burden of MSM disease varies considerably among various regions.

### Global trends by SDI in MSM

High SDI regions continuously had the greatest rates of MSM age-standardized prevalence, incidence, and DALYs throughout 1990 and 2021, whereas low SDI areas had the lowest rates of these indicators (Supplemental Digital Content Fig. S2, available at: http://links.lww.com/JS9/E628). In addition, the middle SDI region had the most dramatic increases in age-standardized prevalence and incidence, with the most noticeable decreases in DALY rates (Table 1, Supplemental Digital Content Table S1, available at: http://links.lww.com/JS9/E780, Supplemental Digital Content Table S2, available at: http://links.lww.com/JS9/E781). Remarkably, the rates of age-standardized DALYs in other regions exhibited a downward trend, while the low-middle SDI region displayed an upward trend. We found that the number of cases rose as the SDI increased, as did the probabilities of prevalence, incidence, and DALYs. In summary, the total burden suggested a growing tendency from 1990 to 2021, despite declining age-standardized DALY rates in the majority of SDI areas. Additionally, there is a favorable correlation between MSM burden and SDI.

### Regional trends associated with SDI in MSM

**Figure 3.**
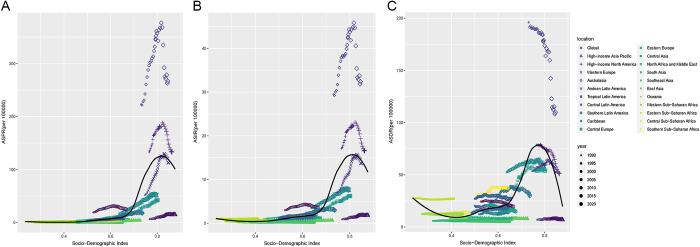
ASPR (A), ASIR (B), and ASDR (C) of MSM for 21 regions by SDI from 1990 to 2021. The expected values based on the SDI and the incidence of disease at all locations are represented by black lines. ASPR, age-standardized prevalence rates; ASIR, age-standardized incidence rates; ASDR, age-standardized DALY rates; MSM, malignant skin melanoma; SDI, socio-demographic index; DALY, disability-adjusted life year.

From 1990 to 2020, this study sheds light on the correlation between SDI and age-standardized rates of DALYs, incidence, and prevalence. These indicators generally exhibit a non-linear connection with the SDI. Age-standardized prevalence and incidence showed a positive correlation with the SDI when the SDI was less than 0.8 and larger than 0.7. As the SDI exceeded 0.8, there was a negative correlation. While age-standardized prevalence and incidence rates were lower than anticipated in high-income Asia Pacific and higher than anticipated in Australasia, they were as predicted in Western Europe (Fig. 3A, B). When the SDI was less than 0.4, there was a negative connection with age-standardized DALYs, and Eastern sub-Saharan Africa had a higher indication than predicted. There was a positive connection between the SDI and age-standardized DALYs when it was higher than 0.6 but lower than 0.8. Tropical Latin America and Southern sub-Saharan Africa had higher SDIs than predicted. The SDI was lower than predicted for high-income Asia Pacific and higher than expected for Australasia, and it had a negative correlation with age-standardized DALYs when it was more than 0.8 (Fig. 3C). Altogether, substantial regional variations in the burden of MSM were involved in variations in SDI.

### National trends in MSM

**Figure 4.**
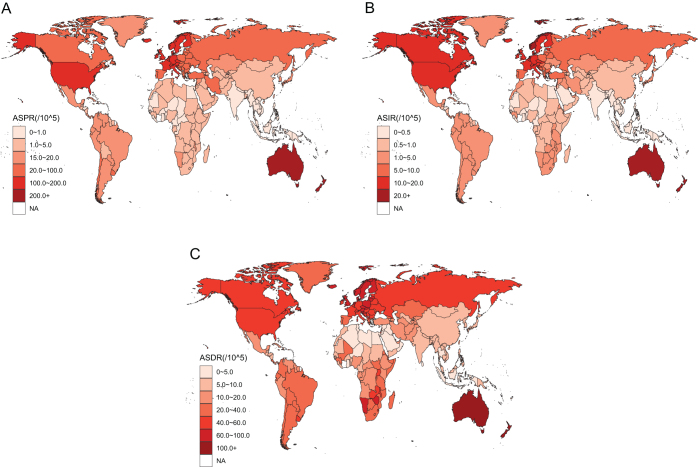
The global ASPR (A), ASIR (B), and ASDR (C) of MSM in 204 countries in 2021. ASPR, age-standardized prevalence rates; ASIR, age-standardized incidence rates; ASDR, age-standardized DALY rates; MSM, malignant skin melanoma; DALY, disability-adjusted life year.

The USA had the greatest MSM prevalence in 2021 at 706 831.05 (95% UI 669 763.82–731 846.16), followed by Germany at 175 636.42 (95% UI 158 866.52–193 437.25), while there were 15 nations with fewer than one case. At 319.03 (95% UI 289.54–348.98), New Zealand had the greatest age-standardized prevalence, while Kiribati had the lowest at 0.06 (95% UI 0.02–0.08). Incidence cases were greatest in the USA (90 444.96; 95% UI 84 956.91–93 999.38) and Germany (22 491.03; 95% UI 20 212.18–24 731.94). The minimal incidence for Tokelau was 0 (95% UI 0–0.01). With an ASIR of 38.91 (95% UI 35.21–42.43), New Zealand had the highest rate, while Sao Tome and Principe had the lowest at 0.03 (95% UI 0.02–0.05). The countries with the highest DALYs were the USA at 263 506.49 (95% UI 247 513.34–280 592.89), China at 153 206.39 (95% UI 82 541.49–204 247), and Russian Federation at 111 183.09 (95% UI 101 641.61–119 962.3), and the lowest was Tokelau at 0.11 (95% UI 0.07–0.19). New Zealand had the greatest age-standardized DALYs (124.64, 95% UI 113.07–135.24), while Sao Tome and Principe had the lowest (0.6, 95% UI 0.33–0.82). The above-mentioned statistics are exhibited in Supplemental Digital Content Table S3, available at: http://links.lww.com/JS9/E782, Supplemental Digital Content Figure S3, available at: http://links.lww.com/JS9/E629, and Figure 4. To summarize, there are significant variations in the worldwide burden of MSM illness among nations.

### Trends in MSM burdens over the next 15 years

**Figure 5.**
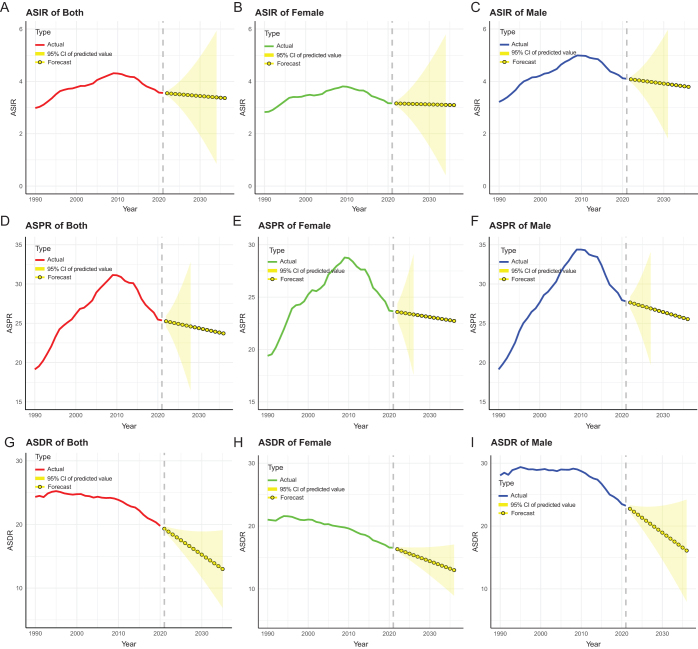
Predicted trends of ASIR (A–C), ASPR (D–F), and ASDR (G–I) of MSM globally over the next 15 years. Red, green, and blue lines represent the actual trend; yellow dotted lines and shaded regions represent the forecast trend and its 95% CI. ASPR, age-standardized prevalence rates; ASIR, age-standardized incidence rates; ASDR, age-standardized DALY rates; MSM, malignant skin melanoma; CI, confidence interval; DALY, disability-adjusted life year.

Over the following 15 years, the worldwide burden of MSM was expected to decline generally, according to the ARIMA model (Fig. 5). Age-standardized incidence, prevalence, and DALY decreased from 3.5/100 000, 25.3/100 000, and 19.7/100 000 in 2021 to 3.6/100 000, 23.1/100 000, and 10.7/100 000 in 2036, respectively. The downward trend in age-standardized prevalence and DALY was more pronounced than in age-standardized prevalence. Furthermore, males exhibited a more marked decline in age-standardized incidence, prevalence, and DALY than females did. This implies that the incidence and prevalence of the disease in females should receive more attention in the future and that specific defenses would need to be implemented. In addition, based on SDI regional stratification, we predicted future trends related to MSM (Supplemental Digital Content Fig. S4, available at: http://links.lww.com/JS9/E630). The data results show that the ASIR, ASPR, and age-standardized disability rate in high SDI and high-middle SDI regions all exhibit varying degrees of decline, while the relevant indicators in the middle SDI, middle-low SDI, and low SDI regions show an upward trend. This suggests that in the future, we need to adopt appropriate response measures for different SDI levels.

## Discussion

Melanocytes are the source of MSM, which can occur at any age but is most common in adults^[1]^. It is deadly and has a high rate of spread^[22,23]^. In this study, we thoroughly investigated research on the prevalence and rate of change of MSM across all age categories in 204 nations, 21 regions, and 5 SDI areas from 1990 to 2021. Based on the GBD 2021 research, this thorough analysis offers important insights into trends in the burden of MSM across various groups and geographical areas, emphasizing the pressing need for more focused and efficient treatments.

### Rising global burden

The research showed that, in comparison to 2019, the age-standardized prevalence and incidence in 2021 increased by 32.98% and 20%, respectively. This implies a progressive escalation in the adverse effects of MSM. Age-standardized DALY rates, however, decreased from 24.3 in 1990 to 19.6 in 2021. It may be possible that improved knowledge of the illness and developments in treatment (such as immunotherapies and targeted medicines) helped minimize the decline in health among MSM sufferers^[24–27]^. The steadily rising worldwide burden of MSM forces us to focus on early detection and prevention from a global standpoint. The development of MSM is caused by several factors, including trauma, irritation, sunshine, race, and heredity^[28–31]^. Thus, it is essential to thoroughly investigate the possible causes of it and reinforce preventative strategies like regular skin screenings and education on sun protection. This aligns with the Global Cancer Observatory (https://gco.iarc.fr/en), which emphasizes early detection through population-based screening and UV exposure reduction campaigns^[32]^. Furthermore, it is imperative to do additional research on the fundamental processes of disease from the viewpoints of genomes and molecular biology with the goal of enhancing early diagnosis and treatment effectiveness.

### Variations by gender

According to our findings, males are more likely than females to have MSM, and the incidence and prevalence of MSM in both sexes are rising, with males experiencing a quicker rate of increase. Additionally, DALYs remained greater in males than in women, despite a declining trend. This implies that male patients have a greater illness burden than female patients. Numerous factors, like chromosome differences, variations in sunshine exposure, and daily life, contribute to the disparity in MSM throughout the sexes^[33–35]^. One of the factors contributing to the increased prevalence of MSM in men may be a lack of knowledge about sun protection and skin care compared to women^[36]^. Special attention is needed given the high frequency of male patients and the DALYs, which may have an impact on patients’ psychological load. Healthcare professionals should also apply gender-specific psychological, therapeutic, and preventative treatments for patients of both genders. For instance, early screening advice and sunscreen teaching need to be reinforced for male patients who work outside more frequently. With national screening programs such as the skin check program run by the Melanoma Institute of Australia, targeted adjustments based on gender further optimize early detection^[37]^. Therefore, to succeed in the medical treatment of the physical and psychological burden of sickness in patients, healthcare professionals ought to offer a variety of comprehensive therapies.

### Regional differences

Analysis of the regions revealed that the MSM burden differed greatly among them. The greatest age-standardized prevalence, incidence, and DALY rates were seen in Western Europe, Australasia, and high-income North America, indicating a substantial MSM burden in these areas. Residents in these regions may be more susceptible to the illness due to their high levels of outdoor exercise and UV exposure. Furthermore, the improved quality of medical treatment in these regions facilitates more effective early identification and reporting of MSM, which raises prevalence rates^[38]^. This is also the reason why the DALYs in these regions are trending downward. On the other hand, the regions with the lowest illness burden are Oceania and Southeast Asia. This could have to do with the dearth of healthcare in the region, which results in underdiagnosis and a failure to show their actual sickness and morbidity^[39]^. Interestingly, these rates grew the greatest in Eastern Europe, suggesting that the MSM burden in this region is rising quickly. This phenomenon may have its roots in the region’s economic and social growth, which has accelerated the burden of MSM by causing changes in lifestyle, greater urbanization, etc. In Western Europe, where the MSM burden is highest, national policies like the EU’s Code Against Cancer already promote sun protection and screening; our data can inform resource allocation within these frameworks. Conversely, in Southeast Asia, aligning with WHO’s “Health System Strengthening” initiative could address underdiagnosis by improving local screening capacity.

### SDI disparities

In low-SDI regions, age-standardized prevalence, incidence, and DALY rates are low and have either slightly grown or declined during the previous three decades. Given the general underdevelopment of healthcare in the area, we suggest that this may be the result of either underreporting or misreporting. Because of their rapid socioeconomic growth, countries with an SDI between 0.7 and 0.8 have a positive association between these variables and the SDI. This also parallels the findings of previous studies, further confirming the reliability of the conclusion^[40]^. The awareness of the population in the region about the prevention of the disease, as well as the overall improvement in medical facilities and standards, has led to an increase in the early diagnosis of MSM^[41]^. However, age-standardized prevalence, incidence, and rates of DALYs were consistently higher in high SDI areas than in other areas. For SDIs higher than 0.8, correlation analysis revealed a negative association with these indicators. Higher SDI areas are more aware of early screening and have better healthcare procedures. In comparison to other places, the burden of MSM may be higher in high SDI areas on account of lifestyle choices, including extensive sun exposure and outdoor activities.

### National burden

The country with the greatest age-standardized prevalence, incidence, and rates of DALYs in 2021 was New Zealand, which was followed by Australia and Norway. Australia and New Zealand are mostly located in the southern latitudes and upper latitudes, where the ozone layer is thinner and the UV light is harsher, making them more vulnerable to illness^[42]^. The majority of people in Norway are Caucasian, making them extremely sensitive to UV rays and vulnerable to acquiring MSM from short-term UV exposure^[43]^. In contrast, African countries such as Ghana and Sao Tome and Principe have low levels of these indicators. This could be because the majority of individuals in these nations have darker skin and greater melanin levels, making them less susceptible to the illness brought on by UV radiation. Furthermore, the general quality of healthcare in these nations is subpar, which leads to insufficient early MSM screening and diagnosis.

### Need for effective interventions

Globally, the burden on various areas and communities in terms of physical and mental health is growing annually due to the rising prevalence and incidence of MSM. Although DALYs are trending to decrease, this does not lessen the total burden strain caused by MSM. This implies that the stress caused by MSM cannot be addressed by the diagnostic and therapy methods we now have and that more focused and customized treatment programs are desperately needed. To lower the occurrence, we should further improve preventative measures and take into account lifestyle, employment type, heredity, and other variables that may contribute to MSM. In economically developed areas, basic research on MSM identifies at-risk people, encourages the development of innovative diagnostic and treatment technologies, and gives tremendous promise for reducing the burden caused by these diseases. However, economically disadvantaged areas must enhance their entire medical infrastructure and standards as well as improve their treatment capacity. By raising public awareness and understanding and advocating for low-cost screening techniques, healthcare professionals may promote early screening. More importantly, in the face of a global disease, there is a need for global collaboration to advance the diagnosis and treatment of MSM. Furthermore, disease trend forecasting is essential for both disease control and prevention. Even if MSM incidence continues to rise, there should be some improvement in prevalence and the overall health effects of the condition in the future. This outcome is consistent with advances in healthcare and heightened awareness of prevention. Nonetheless, there is still a need for enhancements to comprehensive prevention efforts for MSM, particularly for female patients.

### Limitations

Some of the study’s shortcomings cannot be disregarded when evaluating its findings. First, the prevalence of regional socioeconomic disparities may result in inconsistent data collection. Secondly, parameter selections may alter the findings and diverge from the actual scenario when we use the GBD database to develop the model. Thirdly, the accuracy of the analysis may be impacted by the temporal lag of such data. As a result, actual data are required to further validate the findings of this study.

## Conclusions

In summary, over the past three decades, the health burden of MSM has increased worldwide at all ages, with notable variations by nation, gender, and SDI level. To lessen the health burden of MSM, we require more efficient, focused therapies for various groups and geographical areas. Future research should thoroughly investigate the possible risk factors for MSM and develop focused diagnostic and treatment strategies that effectively reduce the global burden of MSM.

## Data Availability

The datasets used during the current study are available from the corresponding author upon reasonable request.

## Research registration unique identifying number (UIN)

Name of the registry: not applicable. Unique identifying number or registration ID: not applicable. Hyperlink to your specific registration (must be publicly accessible and will be checked): not applicable.

## References

[1] Long GV Swetter SM Menzies AM Gershenwald JE Scolyer RA. Cutaneous melanoma. Lancet 2023;402:485–502. 37499671 10.1016/S0140-6736(23)00821-8

[2] Brunsgaard EK Wu YP Grossman D. Melanoma in skin of color: part I. Epidemiology and clinical presentation. J Am Acad Dermatol 2023;89:445–56. 35533771 10.1016/j.jaad.2022.04.056

[3] Adamson AS Suarez EA Welch HG. Estimating overdiagnosis of melanoma using trends among black and white patients in the US. JAMA Dermatol 2022;158:426–31. 35293957 10.1001/jamadermatol.2022.0139 PMC8928089

[4] Pampena R Piccolo V Muscianese M. Melanoma in children: a systematic review and individual patient meta-analysis. J Eur Acad Dermatol Venereol 2023;37:1758–76. 37210654 10.1111/jdv.19220

[5] Hodis E Watson IR Kryukov GV. A landscape of driver mutations in melanoma. Cell 2012;150:251–63. 22817889 10.1016/j.cell.2012.06.024 PMC3600117

[6] Noonan FP Zaidi MR Wolnicka-Glubisz A. Melanoma induction by ultraviolet A but not ultraviolet B radiation requires melanin pigment. Nat Commun 2012;3:884. 22673911 10.1038/ncomms1893 PMC3621412

[7] Anestopoulos I Kyriakou S Tragkola V. Targeting the epigenome in malignant melanoma: facts, challenges and therapeutic promises. Pharmacol Ther 2022;240:108301. 36283453 10.1016/j.pharmthera.2022.108301

[8] Gray-Schopfer V Wellbrock C Marais R. Melanoma biology and new targeted therapy. Nature 2007;445:851–57. 17314971 10.1038/nature05661

[9] Garbe C Peris K Hauschild A. Diagnosis and treatment of melanoma. European consensus-based interdisciplinary guideline - Update 2016. Eur J Cancer 2016;63:201–17. 27367293 10.1016/j.ejca.2016.05.005

[10] Dessinioti C Stratigos AJ. An epidemiological update on indoor tanning and the risk of skin cancers. Curr Oncol 2022;29:8886–903. 36421352 10.3390/curroncol29110699 PMC9689757

[11] Sawada Y Nakamura M. Daily lifestyle and cutaneous malignancies. Int J Mol Sci 2021;22:5227. 34069297 10.3390/ijms22105227 PMC8156459

[12] Agha R Mathew G Rashid R. Transparency In The reporting of Artificial Intelligence – the TITAN guideline. Premier J Sci 2025;10:100082.

[13] GBD 2021 Diseases and Injuries Collaborators. Global incidence, prevalence, years lived with disability (YLDs), disability-adjusted life-years (DALYs), and healthy life expectancy (HALE) for 371 diseases and injuries in 204 countries and territories and 811 subnational locations, 1990-2021: a systematic analysis for the Global Burden of Disease Study 2021. Lancet 2024;403:2133–61. 38642570 10.1016/S0140-6736(24)00757-8 PMC11122111

[14] GBD 2021 Risk Factors Collaborators. Global burden and strength of evidence for 88 risk factors in 204 countries and 811 subnational locations, 1990-2021: a systematic analysis for the Global Burden of Disease Study 2021. Lancet 2024;403:2162–203. 38762324 10.1016/S0140-6736(24)00933-4 PMC11120204

[15] Wang Z Hu L Li J Wei L Zhang J Zhou J. Magnitude, temporal trends and inequality in global burden of tracheal, bronchus and lung cancer: findings from the Global Burden of Disease Study 2017. BMJ Glob Health 2020;5:e002788. 10.1136/bmjgh-2020-002788 PMC7542628 33028698

[16] World Health O. WHO Methods and Data Sources for Country-level Causes of Death 2000-2019. World Health Organization. Geneva. Contract No.: WHO/DDI/DNA/GHE/2020.2; 2020:2020.

[17] GBD 2016 Alcohol Collaborators. Alcohol use and burden for 195 countries and territories, 1990-2016: a systematic analysis for the Global Burden of Disease Study 2016. Lancet 2018;392:1015–35. 30146330 10.1016/S0140-6736(18)31310-2 PMC6148333

[18] GBD 2019 Diseases and Injuries Collaborators. Global burden of 369 diseases and injuries in 204 countries and territories, 1990-2019: a systematic analysis for the Global Burden of Disease Study 2019. Lancet 2020;396:1204–22. 33069326 10.1016/S0140-6736(20)30925-9 PMC7567026

[19] Kim HJ Fay MP Feuer EJ Midthune DN. Permutation tests for joinpoint regression with applications to cancer rates. Stat Med 2000;19:335–51. 10649300 10.1002/(sici)1097-0258(20000215)19:3<335::aid-sim336>3.0.co;2-z

[20] Danpanichkul P Ng CH Tan DJH Wijarnpreecha K Huang DQ Noureddin M. The global burden of alcohol-associated cirrhosis and cancer in young and middle-aged adults. Clin Gastroenterol Hepatol 2024;22:1947–1949.e3. 38428708 10.1016/j.cgh.2024.02.011 PMC11344661

[21] Yang K Yang X Jin C. Global burden of type 1 diabetes in adults aged 65 years and older, 1990-2019: population based study. BMJ 2024;385:e078432. 38866425 10.1136/bmj-2023-078432 PMC11167563

[22] Li C Zhang Q Li Z. Efficacy and safety of carbon-ion radiotherapy for the malignant melanoma: a systematic review. Cancer Med 2020;9:5293–305. 32524777 10.1002/cam4.3134 PMC7402834

[23] Dong Y Wei J Yang F Qu Y Huang J Shi D. Nutrient-based approaches for melanoma: prevention and therapeutic insights. Nutrients 2023;15:4483. 37892558 10.3390/nu15204483 PMC10609833

[24] Pozniak J Pedri D Landeloos E. A TCF4-dependent gene regulatory network confers resistance to immunotherapy in melanoma. Cell 2024;187:166–183.e25. 38181739 10.1016/j.cell.2023.11.037

[25] Long GV Menzies AM Scolyer RA. Neoadjuvant checkpoint immunotherapy and melanoma: the time is now. J Clin Oncol 2023;41:3236–48. 37104746 10.1200/JCO.22.02575

[26] Lazaroff J Bolotin D. Targeted therapy and immunotherapy in melanoma. Dermatol Clin 2023;41:65–77. 36410984 10.1016/j.det.2022.07.007

[27] Ascierto PA Casula M Bulgarelli J. Sequential immunotherapy and targeted therapy for metastatic BRAF V600 mutated melanoma: 4-year survival and biomarkers evaluation from the phase II SECOMBIT trial. Nat Commun 2024;15:146. 38167503 10.1038/s41467-023-44475-6 PMC10761671

[28] Choi ME Cho H Won CH Chang SE Lee MW Lee WJ. Clinicopathologic characteristics of trauma-related nail apparatus melanoma: a comparative study according to the presence of trauma prior to melanoma development. Dermatology 2023;239:165–73. 35878586 10.1159/000525726

[29] Jin SG Padron F Pfeifer GP. UVA Radiation, DNA Damage, and Melanoma. ACS Omega 2022;7:32936–48. 36157735 10.1021/acsomega.2c04424 PMC9494637

[30] Rosenthal A Reddy S Cooper R. Disparities in melanoma-specific mortality by race/ethnicity, socioeconomic status, and health care systems. J Am Acad Dermatol 2023;88:560–67. 36228942 10.1016/j.jaad.2022.10.004

[31] Quek C. Genetics and genomics of melanoma: current progress and future directions. Genes (Basel) 2023;14:232. 36672973 10.3390/genes14010232 PMC9859125

[32] Langselius O Rumgay H de Vries E. Global burden of cutaneous melanoma incidence attributable to ultraviolet radiation in 2022. Int J Cancer 2025;157:1110–19. 40421619 10.1002/ijc.35463

[33] Liu-Smith F Ziogas A. Age-dependent interaction between sex and geographic ultraviolet index in melanoma risk. J Am Acad Dermatol 2020;82:1102–8.e3. 29203439 10.1016/j.jaad.2017.11.049 PMC5984658

[34] Behbahani S Maddukuri S Cadwell JB Lambert WC Schwartz RA. Gender differences in cutaneous melanoma: demographics, prognostic factors, and survival outcomes. Dermatol Ther 2020;33:e14131. 32757248 10.1111/dth.14131

[35] Taglialatela I Indini A Santanelli G. Melanoma and sex hormones: pathogenesis, progressive disease and response to treatments. Tumori 2024;110:309–18. 38372040 10.1177/03008916241231687

[36] Manne S Heckman CJ Kashy D. Moderators of the effects of mysmartskin, a web-based intervention to promote skin self-examination and sun protection among individuals diagnosed with melanoma. Ann Behav Med 2022;56:804–15. 35028656 10.1093/abm/kaab104 PMC9345181

[37] Gordon LG Brynes J Baade PD. Cost-effectiveness analysis of a skin awareness intervention for early detection of skin cancer targeting men older than 50 years. Value Health 2017;20:593–601. 28408001 10.1016/j.jval.2016.12.017

[38] Del Marmol V. Prevention and screening of melanoma in Europe: 20 years of the Euromelanoma campaign. J Eur Acad Dermatol Venereol 2022;36:5–11. 10.1111/jdv.18195 35738812

[39] Harvey VM Patel H Sandhu S Wallington SF Hinds G. Social determinants of racial and ethnic disparities in cutaneous melanoma outcomes. Cancer Control 2014;21:343–49. 25310216 10.1177/107327481402100411 PMC4505912

[40] Li Z Fang Y Chen H. Spatiotemporal trends of the global burden of melanoma in 204 countries and territories from 1990 to 2019: results from the 2019 global burden of disease study. Neoplasia 2022;24:12–21. 34872041 10.1016/j.neo.2021.11.013 PMC8649617

[41] Wainstein A Algarra SM Bastholt L. Melanoma early detection and awareness: how countries developing melanoma awareness programs could benefit from melanoma-proficient countries. Am J Ther 2015;22:37–43. 24914500 10.1097/MJT.0000000000000038

[42] McKenzie R Connor B Bodeker G. Increased summertime UV radiation in New Zealand in response to ozone loss. Science 1999;285:1709–11. 10481002 10.1126/science.285.5434.1709

[43] Bentham G Aase A. Incidence of malignant melanoma of the skin in Norway, 1955-1989: associations with solar ultraviolet radiation, income and holidays abroad. Int J Epidemiol 1996;25:1132–38. 9027516 10.1093/ije/25.6.1132

